# The New Zealand National Trauma Registry: an essential tool for trauma quality improvement

**DOI:** 10.1007/s00068-023-02310-z

**Published:** 2023-07-06

**Authors:** Ian Civil, Siobhan Isles, Alaina Campbell, James Moore

**Affiliations:** 1 Department of Surgery, University of Auckland and National Trauma Network, Wellington, New Zealand; 2 National Trauma Network, Wellington, New Zealand; 3 Waikato Hospital, Hamilton, New Zealand; 4 Intensive Care Unit and Department of Anaesthesia, Wellington Regional Hospital, Wellington, New Zealand

**Keywords:** Trauma, Quality improvement, Registry, Outcomes

## Abstract

**Purpose:**

Trauma registries are essential tools for trauma systems and underpin any quality improvement activities. This paper describes the history, function, challenges, and future goals of the New Zealand National Trauma Registry (NZTR).

**Methods:**

Using the available publications and knowledge of the authors, the development, governance, oversight, and usage of the registry is outlined.

**Results:**

The New Zealand Trauma Network has run a national trauma registry since 2015 and this now contains over fifteen thousand major trauma patient records. Annual reports and a range of research outputs have been published. Key quality improvement initiatives have been undertaken and are described. Vulnerabilities include lack of longterm funding and a small workforce.

**Conclusions:**

The NZTR has proven to be a critical component of trauma quality improvement in New Zealand. A user-friendly portal and a simple minimum dataset have been keys to successes but maintenance of an effective structure in a constrained healthcare system is a challenge.

## Introduction

New Zealand is a small country with a population of just over five million people. It is spread over 1600 km with a land mass of 208,000 sq. km on two main islands. With large agricultural and forestry sectors and the need for considerable distances of road travel, trauma has always been a significant component of healthcare presentations in both urban and rural environments. Medical treatment is provided through a fully funded public healthcare system in hospitals. There are 22 hospitals capable of caring for major trauma patients broadly organised into four regions and in each of these regions there is at least one tertiary trauma hospital (roughly equivalent to an American College of Surgeons Level 1 category). The overarching funding for both acute and long-term trauma care is provided by the Accident Compensation Corporation (ACC), a no fault universal accident insurance organisation.

## History of trauma care in New Zealand

Prior to the 2000s, most trauma care was provided in the hospital closest to the site of injury [1]. Patients were only transferred to a higher level hospital for specific surgical interventions that were not available in the local hospital. Examples would be to a cardiothoracic unit for treatment of a thoracic aortic injury or to a neurosurgical unit for treatment of an epidural hematoma or depressed skull fracture. Although generally satisfactory, this approach to trauma care was associated with mortality rates that were higher than in countries with organised trauma systems and with significant geographic and ethnic inequity.

As a result of national advocacy in the late 2010s by trauma care clinicians, the Ministry of Health agreed to the establishment of the National Trauma Network (NTN) This Network was charged with facilitating optimal trauma care by establishing a formal trauma care system and structure across New Zealand, establishing the NZ Trauma Registry (NZTR), and developing consistent national guidelines. This required definition of prehospital destination policies, encouraging consistent in-hospital care and ensuring progression to appropriate post-injury rehabilitation.

## Current New Zealand trauma system

When an individual suffers trauma requiring medical care, access is obtained by a nationwide single emergency number (111). Prehospital care is provided by either road or helicopter air ambulance services, depending on the triage assessment and location of the patient. Ninety five percent of road ambulance services come from a single provider (St John Ambulance) with the remainder from the Wellington Free Ambulance. Helicopter air ambulance services are regionalised and provided by three regional services (two of the four healthcare regions are served by a single air ambulance service). Each air ambulance service utilises a number of specific helicopter providers in a range of locations.

Using pre-agreed destination policies, approximately 75% of patients are transported directly to a hospital that is capable of providing definitive trauma care and the remaining 25% are subsequently transferred from a first hospital to a definitive hospital. The prehospital destination policies address common prehospital scenarios like serious traumatic brain injury, burns and spinal cord injury and provide guidance and direction so that patients with these conditions are taken directly to the appropriate hospitals [2]. Clinical care is provided at a hospital level generally by one of the surgical specialties (general surgery and orthopaedics most commonly) in the absence of a specific trauma service or surgeons with trauma surgery training. At discharge, options for residential rehabilitation care are limited with only three of the five metropolitan centres having brain injury rehabilitation available locally. Various non-residential rehabilitation options are available in all regions and all are funded by ACC. Patients also receive work-related compensation equivalent to 80% of their normal wage while recovering and in some cases are paid lump sum compensation as well. The overall mortality for major trauma cases ISS (> 12) is around 7% [3] and overall mortality for admitted trauma patients between 2 and 3% [4].

## The New Zealand Trauma Registry (NZTR)

### Who is included in the registry?

The NZTR is a population-based registry which collects information on everyone with major trauma who is admitted to hospital. Inclusion criteria include an ISS > 12 (based on Abbreviated Injury Scale [AIS] 2005 Update 2008) or in-hospital death following injury. Exclusions include external causes which do not result in energy transfer injury, such as hangings, drownings, poisoning, as well as isolated neck of femur fractures and elderly patients with superficial injury and assessed as having advanced frailty [5, 6].

All trauma hospitals are encouraged to enter data on all admitted major trauma patients and while there is no compulsion to do so, there is a small incentive payment to hospitals for each entry.

Approximately 2500 people are entered into the NZTR annually, with an incidence rate of 45–50/100,000 population [7].

### Which information is registered?

Demographic data about the patient includes the unique identifier used in the public health system for all residents and visitors seeking healthcare. The use of identifiable data enables cross-matching with other data sources and provides a useful adjunct to the core registry data. Information about how the injury occurred includes what the person was doing, what transport mode they were using (if applicable), and location. Pre-hospital and hospital data includes time stamping of key points across the patient journey, vital signs, initial blood markers, and access to computed tomography and specific procedures. The length of stay in ICU and hours of mechanical ventilation are calculated.

Long-term patient-reported outcome measures have also been collected on an one year adult cohort to understand their functional status at 6, 12, and 24 months post-injury. This provides a rich source on information about their recovery post-discharge from hospital. Instruments include EQ5D and WHODAS which are recognised internationally and, similar to the AIS coding system, allows benchmarking between jurisdictions.

### Data collection and data quality

Data collection is done by clinicians or data managers trained in AIS and the NZTR. The data are inputted into the single-instance web-based registry available nationally (and via regular data uploads from one region). Demographic information is auto-populated into the registry while other fields require manual input. Most fields have an inbuilt audit tool to reduce logic errors so that, for example, timestamping across the journey of care is sequential.

There is a strong focus on the data quality to ensure the accuracy and consistency of AIS coding in hospitals where coders typically work alone. Quarterly exercises are undertaken with all coders using anonymised cases to discuss coding discordance and challenging coding questions. An inter-rater exercise was undertaken to assess the accuracy of coding in six large hospitals which identified issues such as discordant coding of head and chest injuries [8]. Further measures include the requirement for all coders to undertake refresher training every 3 years, and patients transferred between hospitals are double-coded.

The security of data held in the NZTR is assured through a range of measures including two-factor authentication, control of authorised users, and utilisation of government-approved security infrastructure.

### Registry organization

The National Trauma Network provides oversight of all aspects of the NTR including the contract with Dendrite Clinical Systems^®^ as the vendor and provider of helpdesk and other services, and ad hoc queries from registry users.

A Data Governance Group (DGG) assesses all proposals to use NZTR data to ensure they are appropriate and compliant with New Zealand’s privacy and ethics regulations. Led by an independent chair, the DGG comprises representation from trauma services in each region as well as experts on Māori data sovereignty, biostatistics, and research.

## Obstacles and possibilities of the registry

In order to ensure that the NZTR fully captures the burden of major trauma in the country, data collection capability has been established in all hospitals where trauma patients may present. This includes both larger tertiary centres and smaller rural and regional hospitals, where data collection and injury coding may be the responsibility of a single clinician who is also juggling other clinical duties. This arrangement risks interruptions in data collection and analysis if the data collector leaves their post, underscoring the importance of building additional capacity and resilience into these roles as a key objective of the network.

A variety of sources of data need to be interrogated including ambulance records, emergency department notes, radiology reports, laboratory databases and coronial reports. There is some concern at the duplication of effort in collecting data that are already available within hospital electronic medical records (often in different databases). To address this issue, integrating the registry software with existing data sources has the potential to minimize unnecessary replication of effort.

Currently, the NZTR does not collect data on patients who have died prior to arrival at a hospital. Given New Zealand’s challenging geography and potential for delays in receiving emergency care due to access or weather, this is an important group to consider inclusion in the registry to better represent the full burden of major trauma on the population.

## Comparison to other registries

The National Trauma Network has taken the view that for a registry to function well it must have an achievable minimum dataset (MDS) in terms of the ability of the system to populate those items. As a result the MDS is relatively small compared to many other registries. While the NZTR was initially established using the COLLECTOR^®^ software, a subsequent change was made to a Dendrite^®^ bespoke product. COLLECTOR^®^ is one of the most commonly used trauma registry products in the USA and is used in a number of Australian States for their state-wide trauma registries. Dendrite^®^ has a huge registry portfolio but had not previously been involved in a trauma registry product so to that extent the NZTR is somewhat different from all other registries.

## Examples of recent research activities/quality improvement activities using registry data

The NZTR has provided an accurate repository of data for patients admitted to hospital from July 2015 with severe trauma. Although patient-reported outcome measures have been collected on a specific cohort of these patients they are not part of a continuous process and currently not included in the registry. As a result, the NZTR is largely used retrospectively as a repository to identify patients who have suffered major trauma and then link them with other databases or activities to address a relevant research question. To date much of this research has been epidemiological in relation to patterns of injury. A major theme from one research group has related to equity of both processes of care and outcome [9–17]. New Zealand has an ethnically diverse population and significant geographical diversity. The possibility that care is inequitable on an ethnic, or location basis is of significant concern and the NZTR has been used to evaluate this.

The NTN contracts the New Zealand Health Quality and Safety Commission (HQSC) to provide a data science capability to work with the NZTR data and also to develop, initiate, manage and evaluate quality improvement initiatives in trauma care. The key to formulate and evaluate these quality improvement initiatives have been the data held by the NZTR. To date the HQSC has undertaken specific quality improvement projects focusing on critical haemorrhage, severe traumatic brain injury, post-injury rehabilitation [18–20].

The NZTR data are reported annually and the data science capabilities of the HQSC have been used to develop new tools to risk adjust the data in a local context. Relative risk analysis and funnel plots are both used in the annual reports and risk adjustment elements are described in the annual report.

## Future prospects/perspectives

The NTN plans to transition the NZTR to the more recent AIS 2015 version within the next twelve months, which represents a significant undertaking. Data collectors and coders will need to be retrained in the new system, and further quality assurance activities will be necessary to ensure coding consistency. However, this updated version offers several benefits, including more comprehensive coding of injuries, greater accuracy in predicting mortality and morbidity after injury, and improved ease of use for coders.

Currently, both of the road ambulance providers and two out of the three air ambulance providers use a common electronic patient report system. There is potential for this system to be integrated with the NZTR, allowing fields from prehospital electronic reports to be pushed directly into the registry. This would minimize duplication of effort and potentially reduce the risk of transcription errors. However, such integration is dependent on finalizing technical solutions and data-sharing agreements.

## Conclusions

The NZTR has become an essential element of trauma quality improvement in New Zealand. While a relatively recent innovation, its user-friendly data entry portal and simple MDS has allowed almost 100% completion of all fields even in a highly resource constrained environment. Simple oversight and effective data governance processes have allowed it to become an important resource for trauma research. Ongoing challenges with timely transition of injury coding and software modifications exist as does the process for ensuring an uninterrupted funding stream.
